# Cardiovascular risk factors among women with self-reported infertility

**DOI:** 10.1186/s40738-017-0034-0

**Published:** 2017-04-11

**Authors:** Shruthi Mahalingaiah, Fangui Sun, J. Jojo Cheng, Erika T. Chow, Kathryn L. Lunetta, Joanne M. Murabito

**Affiliations:** 1 Department of Obstetrics and Gynecology, Boston University School of Medicine, 85 East Concord Street, Boston, MA 02118 USA; 2 Department of Epidemiology, Boston University School of Public Health, Talbot 3E, 715 Albany Street, Boston, MA 02118 USA; 3 Department of Biostatistics, Boston University School of Public Health, Crossstown Center, 801 Albany St, Boston, MA 02118 USA; 4 Framingham Heart Study, Massachusetts. Section of General Internal Medicine, Department of Medicine, Boston University School of Medicine, Boston, MA USA

**Keywords:** Infertility, Cardiovascular disease risk, Menstrual cycle length

## Abstract

**Background:**

Amongst women with certain types of ovulatory disorder infertility, the studies are conflicting whether there is an increased risk of long-term cardiovascular disease risk. This paper evaluates the associations of several CVD risk factors among Framingham women with self-reported infertility.

**Methods:**

Women who completed the Framingham Heart Study Third Generation and Omni Cohort 2 Exam 2 (2008–2011), and reported on past history of infertility and current cardiovascular disease status were included in this cross-sectional study. Directly measured CVD risk factors were: resting blood pressure, fasting lipid levels, fasting blood glucose, waist circumference, and body mass index (BMI). Multivariable models adjusted for age, smoking, physical activity, and cohort. Generalized estimating equations adjusted for family correlations. We performed sensitivity analyses to determine whether the association between infertility and CVD risk factors is modified by menopausal status and menstrual cycle length.

**Results:**

Comparing women who self-reported infertility to those who did not, there was an average increase in BMI (β = 1.03 kg/m^2^, 95% CI: 0.18, 1.89), waist circumference (β = 3.08 in., 95% CI: 1.06, 5.09), triglycerides (β = 4.47 mg/dl, 95% CI:−1.54, 10.49), and a decrease in HDL cholesterol (β = −1.60 mg/dl, 95% CI: −3.76, 0.56). We estimated that infertile premenopausal women have an increased odds of obesity (BMI ≥ 30 kg/m^2^) (OR = 1.56, 95% CI: 1.11, 4.49) and diabetes (OR = 1.96, 95% CI: 0.86, 4.49).

**Conclusions:**

BMI and waist circumference were the most strongly correlated CVD risk factors amongst women reporting a history of infertility.

## Background

Infertility is a multifactorial disease with etiologies attributed to male, female, and unexplained factors. It is defined as attempting conception for 1 year without success, or attempting for 6 months or more without success among those 35 years or older [1]. The main causes of female infertility are ovulatory disorders, including polycystic ovary syndrome (PCOS) and primary ovarian insufficiency (POI); tubal abnormalities, including bilateral tubal occlusion (BTO) and tubal damage; endometriosis; hormonal and endocrine disorders, including thyroid and prolactin abnormalities; cervical mucus abnormalities; and structural or acquired genital tract disorders including uterine fibroids [2].

Amongst women with ovulatory disorder infertility (PCOS and POI), some studies demonstrate increased prevalence and long term concern for cardiovascular disease risk [3–6]. Theage at menopause and menopausal status also alter the risk of cardiovascular morbidity and mortality [7–9]. Amongst women with endometriosis, a recent study using the Nurse’s Health Study II cohort found that women with endometriosis had a relative risk of 1.62 (95% CI: 1.39–1.89) for three cardiovascular heart disease end points studied [10].

Aside from ovulatory disorders and endometriosis, the relationship between infertility and CVD risk factors is still unclear. Some recent studies report on the association of infertility history [11], infertility treatment [12] and pre-menopausal cardiovascular disease with no association. However, due to the conflicting literature, there is still concern that women presenting with infertility may be a susceptible population at risk for cardiovascular disease. Given that cardiovascular disease (CVD) is the leading cause of mortality in women in the U.S [13], identifying risk factors, such as infertility or infertility sub-type, that may be associated with CVD would allow for early identification of risk and initiation of risk reduction strategies.

The objective of this paper was to determine the association between infertility and CVD risk factors (CVD RF) among Framingham Heart Study (FHS) women with self-reported infertility. We hypothesized that women self-reporting for infertility would have more severe markers for CVD. We had the opportunity to conduct this investigation in the FHS, a community-based cohort study unselected for disease status and not skewed to an infertility clinic based sample. To our knowledge, this is the first study that employs a cohort with directly measured CVD RF through a research physical examination and laboratory assay.

## Methods

### Study sample

The Framingham Heart Study (FHS) is a longitudinal population-based cohort study of CVD and any associated risk factors in Framingham, Massachusetts [14]. The current study sample was taken from the Exam 2 data of the FHS Third Generation (Gen 3) and Omni 2 cohorts. A detailed methodologic description of cohort enrollment and follow-up is referenced here [15]. In summary, recruitment for the Gen 3 cohort began in November 2001, and inclusion criteria for the cohort required that participants be at least 20 years of age by the first exam cycle and have at least one parent in the Offspring cohort of the FHS. The Exam 2 cycle for this cohort started in May 2008 and finished February 2011. The Omni 2 cohort was recruited from 2003 to July 2005 and was meant to represent a racially/ethnically diverse group that would be at least 10% of the size of the Gen 3 cohort. The Exam 2 cycle for the Omni 2 cohort occurred at the same time as the Gen 3 Exam 2 cycle. This study was approved by the Institutional Review Board at Boston University School of Medicine. Women who were at risk for pregnancy and who responded to the infertility question were included in the analysis (*n* = 1968).

### Assessment of infertility

Infertility was determined by self-report. At the exam, the physician-administered medical history included the following question: “Have you ever tried to become pregnant for ≥ 1 year without becoming pregnant?” If women answered “yes,” they were considered to have infertility.

### Assessment of cardiovascular risk factors

For Gen 3/Omni 2 cohorts, the CVD risk factors that were assessed by physical exam and direct measurement by laboratory analysis in this study are: resting blood pressure (diastolic and systolic), fasting lipid levels (total cholesterol, triglycerides, high-density lipoproteins), fasting blood glucose, waist circumference, and body mass index (BMI). Participants were classified as having hypertension if they had a systolic blood pressure ≥ 140 mmHg, diastolic blood pressure ≥ 90 mmHg, or were taking medication for either high blood pressure or hypertension. Participants were classified as having high total cholesterol if they had fasting serum cholesterol > 239 mg/dl, or taking lipid-lowering medications [16]. Diabetes mellitus was defined by either fasting blood glucose ≥ 126 mg/dL or receiving treatment for diabetes mellitus. The participants brought medications to the exam and the medications were recorded by the physician.

### Covariates

A participant was determined to be a current smoker if they answered “yes” to the question “Have you smoked cigarettes regularly in the last year?” A measure of physical activity known as the physical activity index was calculated from self-report of responses to questions recording the typical number of hours of sleep and light, moderate, and heavy physical activity, and type of physical activity per day (previously described in Kannel WB 1979).

### Statistical analysis

We performed multivariable regression models to characterize the association between infertility and cardiovascular disease risk factors. Logistic regression was used to assess the association between infertility and dichotomous CVD risk factors (hypertension, diabetes, high total cholesterol, and obesity) separately; generalized estimating equations (GEE) were used to account for family correlations. Linear mixed effect regression models were used to assess the association between infertility and continuous CVD RFs (lipids, SBP, DBP, BMI, waist circumference) separately, taking family structure into account. Covariates in the multivariable models included age, smoking status, physical activity index, and cohort (Gen 3 versus Omni 2). Sensitivity analyses were performed excluding all women who were postmenopausal at the time of the exam (*N* = 711), defined as cessation of menses for 1 year or more. For analyses of cycle length, women who were postmenopausal or did not provide information about cycle length were excluded (*N* = 105). We excluded those with missing data in covariates or CVD RFs. All analyses were performed in R statistical software (R Foundation for Statistical Computing; Vienna, Austria).

## Results

Of our study sample of 1968 women, 282 reported infertility. Table 1 highlights selected characteristics: average age was similar among those reporting infertility, 47.3 years (standard deviation (sd) = 7.7), compared to not reporting infertility 46.4 (sd = 9.4). The number of live births, age at menarche, and proportion that had reached menopause were also comparable. The distribution of CVD RFs is also reported in Table 1, with slight differences for those reporting infertility compared to those not with regards to BMI 28.0 kg/m^2^ (sd = 7.0) vs 26.9 kg/m^2^ (sd = 6.1) and total cholesterol 187.8 mg/dL (sd = 35.8) vs 185.7 mg/dL (sd = 33.9).

**Table 1:** Study population characteristics

	Infertility, no		Infertility, yes	
	*N* = 1686		*N* = 282	
Continuous data	Mean	SD	Mean	SD
Age (years)	46.4	9.4	47.3	7.7
Live births (#)	1.6	1.2	1.5	1.2
Age at menarche (years)	12.8	1.6	12.8	1.5
BMI (kg/m^2^)	26.9	6.1	28.0	7.0
SBP (mmHg)	112.2	14.1	112.6	15.3
DBP (mmHg)	71.7	9.1	71.8	8.9
Total Cholesterol (mg/dL)	185.7	33.9	187.8	35.8
HDL (mg/dL)	67.0	17.3	65.7	17.2
Fasting Glucose (mg/dL)	93.1	16.4	92.2	11.7
Physical activity index	35.6	5.0	35.7	5.1
	Infertility, no		Infertility, yes	
Count data	N	%	N	%
Cohort (number from Gen 3)	1538	91.2%	254	90.1%
Current smoking	169	10.0%	19	6.7%
Hypertension	296	17.6%	53	18.8%
CVD	31	1.8%	5	1.8%
Lipid lowering medication	371	22.0%	63	22.5%
Diabetes mellitus	70	4.2%	11	3.9%
Menopausal	601	35.6%	93	33.0%
Cycle > 35 days^a^	60	6.1%	13	7.7%

^a^Among pre-menopausal women who responded

The results of the primary analyses are summarized in Table 2. Our models estimate that women reporting infertility have an average BMI 1.03 kg/m^2^ (95% CI: 0.18, 1.89) higher and an average waist circumference 3.08 (95% CI: 1.06, 5.09) inches larger than women not reporting infertility when adjusting for age, smoking status, physical activity status, and cohort. Similarly, women reporting infertility had 1.43 (95% CI: 1.09, 1.87) times the odds of being obese (BMI ≥ 30 kg/m^2^) compared to women not reporting infertility, after adjustment. We found slightly stronger effects when restricting to premenopausal women (Table 3). Among the premenopausal women, we estimated that the effect size of infertility on HDL is a decrease of 3.2 mg/dl (95% CI: −5.71, −0.74). Likewise, we estimated an effect size of an increase of 6.27 mg/dl in triglycerides among premenopausal women with infertility compared to premenopausal women without infertility. When excluding postmenopausal women, the directionality of the effect sizes changed for diabetes, glucose and total cholesterol. The association between infertility and diabetes increased drastically, from an odds ratio of 0.96 (95% CI: 0.49, 1.88) to 1.96 (95% CI: 0.86, 4.49), and fasting blood glucose for self-reported infertile women became modestly elevated by 0.37 mg/dl (95% CI:−1.63, 2.36). This is likely due to postmenopausal women who are on targeted therapy for glucose control/diabetes. We explored the association between infertility and CVD RFs in a smaller sample of women who also provided information on menstrual cycle length (*N* = 1152). Among women with cycle length ≤35 days, the association between infertility and the risk factors of increased BMI, increased waist circumference, and decreased HDL was similar to the full premenopausal sample, however caution must be exercised in interpretation and generalization as there was a limited number of women with cycle length > 35 days (Table 4).

**Table 2:** Fully adjusted^a^ models for association between infertility and CVD risk factors

	N		Logistic regression estimate	
CVD Risk Factors – dichotomous	No^b^	Yes^b^	OR	95% CI
Hypertension	1619	349	1.07	(0.77, 1.48)
Total cholesterol > 239 mg/dl	1645	322	1.16	(0.83, 1.61)
BMI ≥ 30 kg/m^2^	1465	503	1.43	(1.09, 1.87)
Diabetes	1883	81	0.96	(0.49, 1.88)
			Linear regression estimate	
CVD Risk Factors – continuous	N		β	95% CI
Systolic Blood Pressure (mm Hg)	1968		−0.18	(−1.94, 1.58)
Diastolic Blood Pressure (mm Hg)	1967		−0.05	(−1.22, 1.12)
Total cholesterol (mg/dl)	1965		1.32	(−2.90, 5.53)
HDL cholesterol (mg/dl)	1965		−1.60	(−3.76, 0.56)
Triglycerides (mg/dl)	1965		4.47	(−1.54, 10.49)
Glucose (mg/dl)	1965		−1.26	(−2.94, 0.41)
BMI (kg/m^2^)	1968		1.03	(0.18, 1.89)
Waist circumference (inches)	1967		3.08	(1.06, 5.09)

^a^Fully adjusted model includes covariates age, smoking status, physical activity index, and cohort

^b^Counts within the entire study population

**Table 3:** Fully adjusted^a^ models for association between infertility and CVD risk factors in the pre-menopausal subset

	N		Logistic regression estimate	
CVD Risk Factors – dichotomous	No^b^	Yes^b^	OR	95% CI
Hypertension	1119	138	0.93	(0.55, 1.55)
Total cholesterol > 239 mg/dl	1157	99	0.85	(0.48, 1.51)
BMI ≥ 30 kg/m^2^	965	292	1.56	(1.11, 2.21)
Diabetes	1225	30	1.96	(0.86, 4.49)
			Linear regression estimate	
CVD Risk Factors - continuous	N		β	95% CI
Systolic Blood Pressure (mm Hg)	1257		−0.87	(−3.11, 1.36)
Diastolic Blood Pressure (mm Hg)	1256		0.03	(−1.51, 1.56)
Total cholesterol (mg/dl)	1255		−1.29	(−5.87, 3.28)
HDL cholesterol (mg/dl)	1255		−3.23	(−5.71, −0.74)
Triglycerides (mg/dl)	1255		6.27	(−1.32, 13.86)
Glucose (mg/dl)	1255		0.37	(−1.63, 2.36)
BMI (kg/m^2^)	1257		1.35	(0.28, 2.43)
Waist circumference (inches)	1256		3.64	(1.11, 6.16)

^a^Fully adjusted model includes covariates age, smoking status, physical activity index, and cohort

^b^Counts within the entire study population

**Table 4:** Fully adjusted models for association between infertility and CVD risk factors stratified by cycle length

Cycle Length ≤35		Linear regression estimates	
CVD Risk Factors - continuous	N	β	95% CI
Systolic Blood Pressure (mm Hg)	1079	−0.65	(−3.13, 1.83)
Diastolic Blood Pressure (mm Hg)	1078	0.20	(−1.53, 1.94)
Total cholesterol mg/dl	1077	0.38	(−4.68, 5.44)
HDL cholesterol mg/dl	1077	−3.66	(−6.35, 0.97)
Triglycerides mg/dl	1077	8.15	(−0.43, 16.72)
Glucose mg/dl	1077	0.73	(−1.43, 2.89)
BMI (kg/m^2^)	1079	1.47	(0.27, 2.68)
Waist circumference (inches)	1078	3.78	(1.01, 6.55)

## Discussion

This study represents a unique application of the Framingham Heart Study in evaluating the cross-sectional association between infertility and cardio-metabolic disease risk factors. To our knowledge, this is the first study that employs a cohort with directly measured CVD RF outcomes through a physical examination and laboratory assay. In this analysis using FHS participants, the prevalence of infertility was 14.2%, which is similar the figure reported in the 2002 National Survey of Family Growth, 8–12% [17]. In our study, for the women self-reporting for infertility, the odds of having diabetes and an obese BMI were higher than their 1686 counterparts not reporting infertility. We also detected a trend towards abnormal lipid parameters, with an elevation in triglycerides and lower serum HDL in the infertile group, further suggesting an association between self-reported infertility with CVD risk factors. The effect size is small, which makes it uncertain whether the presence of infertility is associated with a small increase in CVD risk metrics for all infertile women, or if a subset carries a disproportionate risk. However, this study limited in its ability to evaluate subpopulations due to a small sample size.

The relationship between female factor infertility subtypes (such as ovulatory disorders, endometriosis, and uterine fibroids) and cardiovascular disease risk may be mediated by different pathways. For example, women with PCOS have greater prevalence of elevated total cholesterol, triglycerides, LDL, as well as lower HDL [18–22]. Increased BMI is well-documented in the three hyperandrogenic Rotterdam phenotypes [23, 24], as is the metabolic syndrome [25, 26]. Daan et al. previously noted that the cardiometabolic profile is worse in the hyperandrogenic phenotypes [27]. Thus, the pathway from infertility to CVD may be mediated by hyperandrogenism, dyslipidemia, obesity, and insulin resistance [28]. Further bolstering the possible implications of PCOS on cardiovascular disease risk, a study using the prospective arm of the Women’s Health Initiative employed retrospectively-reported reproductive health outcomes to refine a post-menopausal cardiovascular risk factor model. The study noted that “always having irregular menses” and “sometimes having irregular menses” were positively associated with coronary heart disease, while a history of infertility or cause of infertility were not independently associated with postmenopausal CHD in their model [11]. Thus, it may be that among our study participants self-reporting for infertility, only those with menstrual irregularity carry a greater risk for CVD. One limitation of the study is that they did not evaluate pre-menopausal cardiovascular events and RFs remotely and retrospectively ascertained history of infertility and infertility cause.

Women with diminished ovarian reserve share a similar inclination towards dyslipidemia as women with PCOS, showing increased levels of triglycerides and LDL, and decreased levels of HDL; markers of insulin resistance like HOMA-IR are also elevated, as well as C-reactive protein, a marker of inflammation [29]. Consequently, among women with premature ovarian insufficiency, the pathway to CVD may be mediated by dyslipidemia [7, 8], chronic inflammation [30], and insulin resistance.

Some earlier studies on women with endometriosis show that serum HDL and LDL are similar to that of age- and adiposity-matched controls, but serum lipoprotein (a) and triglycerides are elevated [31]. The relationship between endometriosis and known CVD risk factors is complicated by the fact that women with endometriosis have a lower BMI compared to controls [32, 33] and levels of fibroblast growth factor-2 (FGF-2) are elevated in women with endometriosis [34], since FGF-2 is known to protect against myocardial dysfunction [35]. Endometriosis may lead to CVD through pathways involving dyslipidemia, hysterectomy/oophorectomy [10], and chronic inflammation.

One major cardiovascular risk factor associated with the development of uterine fibroids is hypertension [36]. Biological mechanisms between hypertension and fibroids remains elusive, but may be due to hypertension induced tissue-changes at the level of the myoma cells or at the level of promoting vascular proliferation feeding the myoma.

Obesity, defined as a BMI > 25 kg/m^2^, is associated with increased time to pregnancy couples [37, 38], poorer *in-vitro* fertilization outcomes [39, 40], and also associated with ovulatory disorder in females [41]. In regularly ovulating women, obesity is associated with a longer time to pregnancy [42, 43]. Obesity is known longstanding risk factor for coronary heart disease [44], as well as CVD RFs [45] including diabetes [46, 47], hypertension [48], and dyslipidemia [49].

Our study somewhat contrasts with one by Parikh et al. in 2012. They also conducted a study agnostic to the cause of infertility, instead focusing on the association between the degree of subfertility and incidence of CVD among women in Sweden. Even without adjusting for BMI, they found no association between the degree of subfertility and incidence of CVD among women who were moderately infertile (up to 4 years of involuntary childlessness); only the most extreme infertile subjects (5+ years of involuntary childlessness) had an increased risk of CVD [50]. However, our study agrees with a study based on the Japan Nurses’ Health Study, which showed that Japanese women with ovarian infertility were at high risk of hypercholesterolemia and diabetes mellitus before age 45 [5]. The group with ovarian infertility were also more likely to be obese than the controls.

Our study has some notable limitations. The ascertainment of infertility is through self-report, and thus we may have had subject misclassification. We neither had information regarding work-up or cause of infertility, nor information on any fertility treatments used. Age at diagnosis, time since diagnosis, and treatment of infertility may be relevant to CVD RF development and were not available for the present cross-sectional analysis. Furthermore, we were limited by small sample sizes in our sensitivity analyses for menstrual cycle length > 35 days.

One concern is that the use of fertility treatment, and not the infertility itself, confers CVD risk. Several treatment options are available and have been utilized by women with subfertility and infertility including superovulation drugs (clomiphene citrate and injectable gonadotropins) combined with intrauterine insemination or in-vitro fertilization. Studies regarding the association of infertility treatment with long-term cardiovascular and metabolic risk in women are inconsistent. One study of 23,498 women in Sweden found that women who underwent in-vitro fertilization had an increased risk of hypertension and stroke [51], while another study of 1,186,753 women in Ontario, Canada found that women who delivered after undergoing fertility therapy were not at increased risk of cardiovascular disease [52]. However, there are no published prospective cohort studies that have determined the association of infertility and use of infertility treatment on long-term cardiovascular risk in women.

Our study has several notable strengths. First, there is excellent CVD RF outcome ascertainment with study staff (trained physicians and medical technicians) administered physical exam and study laboratory assessment for CVD RF. This reduces the bias of self-reported CVD RFs noted in other studies. Secondly, this community based cohort may be more representative of the general population compared to hospital or clinic-based cohorts, providing greater generalizability than studies drawn from clinical encounters alone.

## Conclusions

In this study, we found that BMI and waist circumference were the CVD risk factors most strongly correlated with a reported history of infertility among women in their reproductive and menopausal years. Future studies should evaluate CVD RFs in women remote from diagnosis and treatment of infertility, and by infertility sub-type, to better delineate this association.

## Abbreviations

BMI: Body mass index
BTO: Bilateral tubal occlusion
CVD RF: Cardiovascular disease risk factor
CVD: Cardiovascular disease
DAG: Directed acyclic graph
FGF-2: Fibroblast growth factor-2
FHS: Framingham Heart Study
GEE: Generalized estimating equations
Gen 3: Framingham Heart Study Third Generation Cohort
PCOS: Polycystic ovary syndrome
POI: Primary ovarian insufficiency
SD: Standard deviation
